# Simulation and Non-Invasive Testing of Vinegar Storage Time by Olfaction Visualization System and Volatile Organic Compounds Analysis

**DOI:** 10.3390/foods10030532

**Published:** 2021-03-04

**Authors:** Hao Lin, Jinjin Lin, Benteng Song, Quansheng Chen

**Affiliations:** School of Food and Biological Engineering, Jiangsu University, Zhenjiang 212013, China; linhao@ujs.edu.cn (H.L.); 2221818018@stmail.ujs.edu.cn (J.L.); 2221418032@stmail.ujs.edu.cn (B.S.)

**Keywords:** vinegar, non-invasive-testing, olfaction visualization system, volatile organic compounds (VOCs), COMSOL simulation

## Abstract

An olfactory visualization system conducts a qualitative or quantitative analysis of volatile organic compounds (VOCs) by utilizing the sensor array made of color sensitive dyes. The reaction chamber is important to the sensor array’s sufficient and even exposure to VOCs. In the current work, a reaction chamber with an arc baffle embedded in the front of the air inlet for drainage effect was designed. The velocity of field and particle distribution of flow field in the reaction chamber was simulated by COMSOL Multiphysics. Through repeated simulation, the chamber achieved optimal result when the baffle curvature was 3.1 and the vertical distance between the baffle front end and the air inlet was 1.6 cm. Under the new reaction chamber, principal component analysis (PCA) and linear discriminant analysis (LDA) were employed to identify vinegar samples with different storage time through analyzing their VOCs. The LDA model achieved optimal performance when 8 principal components (PCs) were used, and the recognition rate was 95% in both training and prediction sets. The new reaction chamber could improve the stability and precision of an olfactory visualization system for VOCs analysis, and achieve the accurate differentiation and rapid discrimination of Zhenjiang vinegar with different storage time.

## 1. Introduction

The quality of aromatic vinegar is closely related to its storage time. With changes in flavor, composition and content, volatile organic compounds (VOCs) (such as pyrazine, oxazole heterocyclic compounds) of vinegar will increase significantly with time [1,2]. Recently, the study of vinegar volatile odor detection has developed into a relatively independent field of study. The VOCs analysis technologies have wide application, and volatile components in Zhejiang rosy vinegar were analysed by headspace-solid phase microextraction gas chromatography–mass spectrometry (HS-SPME/GC–MS) [3], phenolic compounds in the sugarcane vinegar were analyzed by ultra-high pressure liquid chromatography tandem mass spectrometry (UPLC-MS) [4], aroma extract dilution analysis in the Shanxi vinegars before and after aging were both analyzed by GC-MS and gas chromatography-olfactometry (GC-O) [5], and the volatomic signature of Tinta Negra wines were analysis by liquid-liquid microextraction combined with gas chromatography–ion trap mass spectrometry (LLME/GC–ITMS) [6], etc. These methods, however, usually are complicated, time-consuming or costly, thereby necessitating a non-destructive and fast techniques, such as electronic tongue and electronic nose-based [7], whereby efficient non-destructive techniques are used to simplify the process of distinguishing different samples, although the reliability and accuracy of their results was challenged. Rakow and Suslick [8] firstly introduced the utilization of sensor array in the olfactory visualization system by exposing VOCs to be detected. This olfactory visualization system was convenient, rapid and precise in characterizing VOCs.

In the olfactory visualization system, the reaction chamber is the place where the gas exposed to the colorimetric sensor array (CSA) is measured. The distribution of the gas to be measured in the reaction chamber would directly affect the stability and repeatability between the sensor array and the gas [9]. Su-Yeon Kim [10] established reaction chamber that was a simple cuboid to distinguish different roasting and brewing coffees. In the above studies, most of the design for the reaction chamber is simple, while there was little relevant theoretical support. At present, research on the reaction chamber is gradually increasing. Lin, Yan, Song, et al. [11] designed a reaction chamber of the measured gas and sensor array point. In their research, COMSOL Multiphysics software (COMSOL) was utilized for flow field analysis, and the results showed that the planar contact reaction chamber had better performance. This made the design of the reaction chamber more rigorous in theoretical support.

In view of the above studies, this current work has focused on the design and optimization of the reaction chamber in the olfaction system. The main improved method was to embed an arc baffle. The aim is to distribute the gas slightly and more evenly before the CSA exposure to the gas. In this study, COMSOL was utilized to simulate the flow and separation of the gas in the reaction chamber, which can observe whether the distribution of the gas in the area of the CSA was even or not. COMSOL, a major software for advanced numerical simulation is widely used in engineering calculation and scientific research in various fields [12,13,14]. It is suitable for the coupling of multiple physical fields, such as gas-flowing [15]. Therefore, COMSOL can calculate, and simulate various physical phenomena that can be expressed by partial differential equations, and accurately obtain numerical simulation models of any multiple physical fields [16]. Therefore it can optimize the shape and position of a baffle in the reaction chamber, and finally obtain the optimal solution. The new reaction chamber can be designed for more even VOCs and further characterize the storage time of vinegar.

## 2. Materials and Methods

### 2.1. Samples Preparation

Aromatic vinegar, is local specialty of Zhenjiang, produced by Jiangsu Hengshun Vinegar Industry Co., Ltd. (Jiangsu, China). All samples were classified according to storage time. Three types of vinegar sample were collected based on storage time, those were 1 year, 2-year, and 3-year. Before each sampling, vinegar was fully mixed; 10 mL vinegar was taken from each sample into a 50 mL beaker. A total of 60 samples were collected for further analysis: 20 samples from each type.

### 2.2. Olfactory Visualization System

Bromocresol green is a pH indicator with sensitivity to organic acids and constancy of the spots [17,18]. Bromocresol green indicator and anhydrous ethanol and used in the test were purchased from Sinopharm Chemical Reagent Co., Ltd. (Shanghai, China). The production process of the CSA was as follows: bromophenol green indicator was dissolved in anhydrous ethanol and its capacity fixed to 5 mL in a volumetric flask. To ensure complete dissolution of the solution with a concentration of 2 mg/mL, was exposed to ultrasound for 15minutes. Colorimetric dyes were printed on the anti-phase silicon sheet (Merck Millipore, Darmstadt, Germany) using 100 × 0.3 mm micro-capillary pipettes, constructing a 2 × 2 sensor array.

**Figure 1:**
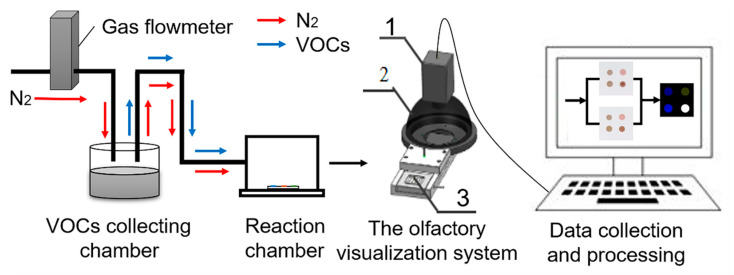
Diagram of the artificial olfaction system. 1. 3 Charge Coupled Device (CCD) camera; 2. Diffuse reflecting light-emitting diode (LED) ball integral light source; 3. Card slot (place colorimetric sensor array).

As shown in Figure 1, The olfactory visualization system mainly includes: (1) 3 Charge Coupled Device (CCD) camera (JAICV-M9GE), which is used to capture the image; the light can be divided into red, green, and blue primary colors, then passed through 3 independent CCD sensors for processing to ensure the authenticity of color. (2) Diffuse reflecting light-emitting diode (LED) ball integral light source (OPT-RID150); it reflects the light from the bottom at 360 degrees, which can ensure that the whole image is uniform. (3) Card slot; a removable slot. (4) Computer; it is the signal output system, which included JAI Camera Control Tool (CV-M9GE) to capture the initial image of the CSA, ACDSee 20 software to process the initial image, and MATLAB R2014a to extract RGB values and further analysis.

### 2.3. Design of Reaction Chamber

**Figure 2:**
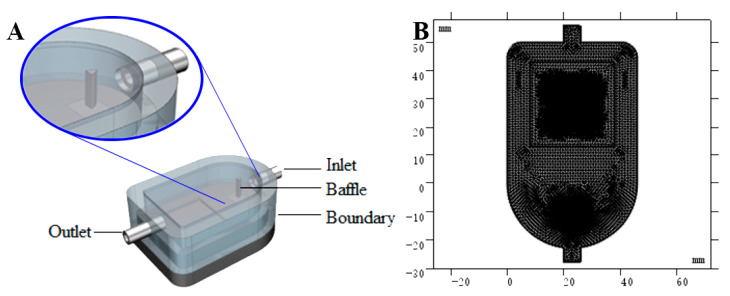
Design of reaction chamber. (**A**) Design of reaction chamber in 3D; (**B**) Grid generation in 2D.

In order to design and optimize the reaction chamber of the olfactory visualization system, this current work intends to embed an arc baffle at the front of the reaction chamber functioning as a diversion, as shown in Figure 2A. The design needs to be optimized in terms of baffle curvature and position of the baffle [19]. In order to illustrate the rationality of the design, the COMSOL was utilized to solve the problem of physical field solution by: (1) solving the problem of partial differential equations to achieve physical field simulation, (2) establishing the two-dimensional model of reaction chamber, and (3) simulating the distribution of gas flow in the reaction chamber with the baffle. At the same time, curvature of baffle and position of baffle embedded in the reaction chamber were constantly changed to summarize the gas law of flow field change in the reaction chamber. In order to meet the requirement of uniform distribution of gas in the reaction chamber, the shape and position of the baffle were constantly optimized.

In order to simplify computation in this study, two dimensions (2D) were utilized as modeling rather than three dimensions (3D) while assuring the validity of simulation as much as possible. Therefore, the Navier–Stokes Equation (1) can be described to acquire the compressible formula for continuity as:

(1) ∂ρ/∂t+∇(ρ·u)=0

And the momentum Equation (2):

(2) $$\rho \partial u/\partial t+\rho \left(u\cdot \nabla \right)u=\nabla \cdot \left[-pI+\mu \left(\nabla u+{\left(\nabla u\right)}^{T}\right)-2/3\mu \left(\nabla \cdot u\right)I\right]+F$$

Here, ρ: density of the fluid (International System of Units (SI): kg/$m^{3}$), u: velocity vector (SI: m/s), μ: dynamic viscosity (SI: Ns/$m^{2}$), p: the pressure (SI: Pa), I: unit tensor, and F: a body force term (SI: N/$m^{3}$). Some parameters need to be indicated as follows: initial transmission velocity ($u_{0}$) of fluid the medium(air) is 0.4 m/s, a velocity vector perpendicular to the boundary at the inlet is $u\cdot n=u_{0}$, the initial pressure ($p_{0}$) is equal to the pressure (p) at the exit boundary that is $p=p_{0}$. For the last one, the velocity at the surface of the sides and the baffle in the reaction chamber were set to zero, which means the boundary is smooth enough, that is u=0.

Grid generation is a very significant part in a COMSOL simulation experiment. The precision and quality of grid generation will directly affect the accuracy of simulation results as discussed by Britz, et al. [20]. In this study, this involved setting the sequence type of grid as user control in the model developer of the grid tool, retaining the size and free generation of a triangular grid in the expansion tool. Next, fluid mechanics need to be selected in the calibration of the size pull-down menu, and then predefined grids selected and the precision set to be more detailed. The other parameters of the freely dissected triangle grid utilize default parameters. Finally, the grid is clicked to implement all the constructions, and the grid can be divided into triangles. The schematic grid generation is shown in Figure 2B.

### 2.4. Classification of Vinegar Samples

According to the simulation results of particle velocity and distribution when VOCs exposed to sensors by COMSOL, a new reaction chamber was designed through embedding an arc baffle in the front of the inlet. Finally, the new reaction chamber applied in the real experiment to classified aromatic vinegar, that is non-invasive classification of vinegar storage time by VOCs and multivariate analysis.

All experiments were carried out in the environment of two types of reaction chamber. The collection and processing of experimental data were as follows: first, before the sensor array was exposed to the gas to be measured, a 3 CCD camera in the olfactory visualization system was used to capture the CSA to in order to obtain the initial image of the CSA. Then, 10mL of balsamic of vinegar was collected in the air collecting chamber with pipette volume, and embed the CSA in the reaction chamber. The gas flowmeter was then opened to extract the gas from the collection chamber into the reaction chamber. The CSA was exposed to VOCs for 15 min [21]. Finally, when the time is up (after the VOCs from vinegar fully exposed to the sensor array), the gas flowmeter was closed and the image of CSA from olfactory visualization system was obtained.

For images of before and after response from the same CSA, RGB (red, green, and blue) values model was performed to obtain the difference value so as to get the total number of variables of 12 (4dot (labeled S1, S2, S3, and S4) ×∆R, ∆G, ∆B value). The value obtained was used as the original data for pattern recognition and analysis [22,23]. For classification, PCA and LDA were used to distinguish vinegar of different storage time [24]. In this work, 60 vinegar samples (3 years and 20 samples each year) were prepared for experiment. Two-thirds (40) of them were taken as the training set and the rest of the one-third (20) as the prediction set for modelling. The experiment was repeated three times in each reaction chamber. MATLAB R2014a was the data analysis software used (Mathworks Inc., Natick, MA, USA).

## 3. Results and Discussion

### 3.1. Simulation Results of COMSOL Software

**Figure 3:**
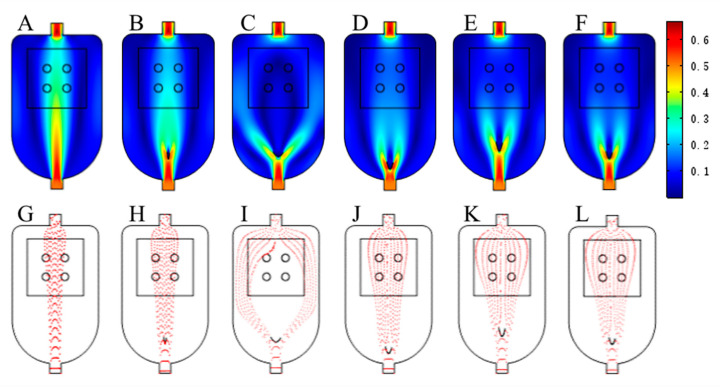
Simulation results of reaction chambers with different conditions. Velocity fields: (**A**). the chamber with a large-curvature; (**B**). the chamber with a small-curvature; (**C**). a baffle close to the inlet; (**D**). a baffle far from the inlet; (**E**). the chamber with an optimized baffle; (**F**). the non-baffle chamber baffle. Particle distribution: (**G**). the chamber with a large-curvature; (**H**). the chamber with a small-curvature; (**I**). a baffle close to the inlet; (**J**). a baffle far from the inlet; (**K**). the chamber with an optimized baffle; (**L**). the non-baffle chamber.

When COMSOL simulation was conducted, the initial curvature range of the baffle was set to 0–10, and the initial range of the baffle front end vertical distance from the inlet was 0.5–2.5 cm. The results of the reaction chamber simulation were divided into velocity field distribution diagram and particle distribution diagram. In the Figure 3, the color ranges from dark blue to dark red represent the gas velocity range from low to high. As the color reaction between the CSA and VOCs only occurs in the CSA region, the flow field uniformity of the region where the CSA was located can be used as the evaluation criteria for the result. In order to fully and uniformly expose the CSA to the VOCs, the molecules of VOCs should be evenly dispersed in the CSA region and pass through the CSA region at a uniform speed as far as possible. Furthermore, in Table 1, velocity data of four color-sensitive dyes areas in the flow field diagram under different velocity fields, will be used to further quantitative evaluate the uniformity and performance. 

#### 3.1.1. The Effect of Baffle Curvature on the Gas Distribution in the Reaction Chamber

Figure 3A,B,G,H show the velocity of field distribution and particle distribution of the VOCs when the baffle curvature was less and larger. It can be seen that the baffle curvature was the key factor influencing VOCs distribution. When the curvature of the baffle was too large, the VOCs flow rate in the middle of the reaction chamber was large, and the gas flow rate on both sides was small. In addition, the gas was mainly distributed over the middle passage region. The gas distribution around the baffle was less, resulting in an uneven flow field. When the curvature of the baffle was too small, the situation will be reversed. When the gas flow rate in the center of the reaction chamber was small, the gas flow rate on both sides of the reaction chamber were large, and the gas was greatly shunted by the baffle.

#### 3.1.2. The Effect of the Position of the Baffle on the Gas Distribution in the Reaction Chamber

Figure 3C,D,I,J indicate that the velocity of field distribution and particle distribution of the gas when vertical distance was smaller and larger between baffle and inlet. When the baffle was close to the inlet, the gas flow rate on both sides of sensor array became smaller. When the baffle was far from the inlet, the gas velocity in the middle of CSA became smaller. At the same time, the particle distribution map shows that when the baffle and air inlet were far apart, the gas distribution near outlet was found to be broader. In summary, the position of baffle may affect distribution of air flow in reaction chamber, but compared with the baffle curvature, this influence was not so good.

#### 3.1.3. The Effect of Reaction Chambers with a Baffle and without a Baffle

After repeated simulation, this study finally determined that when the baffle curvature was 3.1 and the vertical distance between the baffle front end and the air inlet was 1.6 cm, the simulation result was found to be the best. The new reaction chamber after optimization was compared with common reaction chamber without baffle, and the result was obvious visually. Figure 3E,F,K,L show the gas velocity field distribution and particle distribution of the optimized new reaction chamber and the baffle-free reaction chamber. When the shape and position of the baffle were optimized, the gas flow rate of the reaction chamber, especially in part of the CSA, was relatively uniform, and the main part of gas can cover the area of the CSA in general. In the non-baffle reaction chamber, the flow velocity between the two sides was too large, and the flow velocity on both sides was too small. The gas mainly flows in the inlet and outlet direction, and flow field distribution were not uniform, which was not conducive to the uniform reaction between the CSA and the VOCs.

#### 3.1.4. Data Analysis of Experiment

In order to obtain the quantitative analyses of simulation results, COMSOL was utilized to extract the specific velocity values of four color-sensitive dyes areas in the flow field diagram, and the standard deviation (SD) of each area’s velocity was calculated. Table 1 indicates the velocity data of the CSA in the reaction chamber under various simulation schemes. It can be seen that the velocity distribution of reaction chamber after the shape and position optimization of the baffle was average, the flow velocity was 0.116 ± 0.013 m/s, which can better guarantee the uniform reaction of all points of the CSA in the reaction chamber. To sum up, the reaction chamber with an optimized baffle shape and position found the best choice for the experiment.

### 3.2. The Determination Results and Analysis of Vinegar Storage

The response values of colorimetric dyes exposure to Zhenjiang aromatic vinegar with different ages are shown in Table S1, and were further used to model with.

#### 3.2.1. Principal Component Analysis (PCA) under Two Types of Reaction Chamber 

PCA, a linear and unsupervised statistical method and pattern recognition technique, transforms multivariable matrix of raw data set into a set PCs [25]. The 3D images of first three PCs can show the clustering trend of different categories clearly [26].

**Figure 4:**
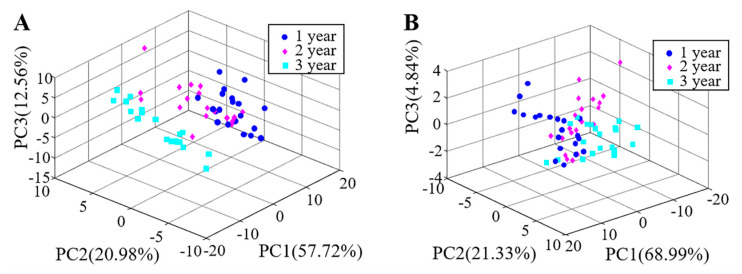
Classification results by principal component analysis (PCA) in different reaction chambers. (**A**). The new reaction chamber; (**B**). The free gas volatile reaction chamber.

Figure 4 shows that distribution of vinegar samples tested in new reaction chamber and free gas volatile reaction chamber respectively of the three-dimensional principal component diagram. The cumulative contribution rate of the first three principal components were 91.26% (PC1 = 57.72%, PC2 = 20.98%, PC3 = 12.56%) and 95.16% (PC1 = 68.99%, PC2 = 21.33%, PC3 = 4.84%) respectively, which could represent majority of information in the characteristic data. It can be seen from the 3D principal component diagram that all of the three vinegar samples have a certain clustering trend in Figure 4, but there were still a small number of samples with overlapping patterns in the various vinegar-aged aromatic vinegar samples. So PCA was difficult to completely distinguish vinegar samples of different storage times.

#### 3.2.2. Linear Discriminant Analysis (LDA) under Two Types of Reaction Chamber

The LDA model maximizes the ratio between group distance and group distance of data set by searching for linear transformation [27]. In this study, LDA classifier was calculated based on obtained principal component variables.

**Figure 5:**
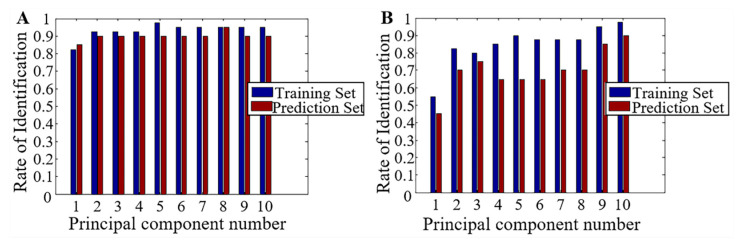
Classification results by linear discriminant analysis (LDA) in different reaction chambers. (**A**). The new reaction chamber; (**B**) the free gas volatile reaction chamber.

LDA was performed for the first 10 PCs. Figure 5 and Table 2 show the LDA discrimination analysis results in the new reaction chamber and free gas volatile reaction chamber, respectively. Under the new reaction chamber, at PCs variable 8, the recognition rate of the training set and prediction set of the model were both 95%. In the free gas volatile reaction chamber, at PCs 10, 97.5% of the vinegar samples in the training set of the model were successfully identified, and 90% of vinegar samples in the predicted concentration were correctly identified. Therefore, the former’s discrimination against vinegar storage was found to be better. Overall, when PCs were same, the olfactory visualization system generally had higher recognition rate for all kinds of storage vinegar samples in new reaction chamber than the free gas volatile reaction chamber, which indicated that model stability and accuracy of the former was higher.

### 3.3. Discussion

This study designed an embedded in front of the inlet arc baffle with distribution effect of reaction chamber, utilizing COMSOL to simulate reaction chamber flow field. The simulation results showed that baffle curvature was a key factor affecting air flow distribution in the reaction chamber, where baffle position also affected air flow distribution. When the curvature of the baffle was too large, the gas flow rate in the middle of the reaction chamber was large, and the gas flow rate on both sides was small. In addition, the gas was distributed mainly over the middle passage region. The gas distribution around the baffle was less, resulting in an uneven flow field. When the baffle curvature was too small, the situation reversed. The gas flow rate in the middle of the reaction chamber was small, and the gas flow rate on both sides was large. The gas greatly shunted from the baffle, which was mainly passed in and out of the reaction chamber twice, while distribution in the middle of the reaction chamber was relatively rare. When the baffle was close to the inlet, gas flow rate on both sides of sensor array became smaller. However, if the baffle was far away from outlet, the gas velocity in the middle of the sensor array became smaller. Meanwhile, the gas distribution near the outlet was broader. Through repeated simulation, this study determined that when the baffle curvature was 3.1 and the vertical distance between the baffle front end and air inlet was 1.6 cm, the system may obtain optimal results. Furthermore, storage time of vinegar was detected and distinguished by the olfactory visual system and vinegar as a representative of liquid food. The identification accuracy of vinegar storage can reach more than 90% through the detection of vinegar VOCs in different storages, coupled with PCA and LDA.

## 4. Conclusions

In this work, a reaction chamber embedded with a baffle was designed to improve the uniformity of the VOCs of vinegar exposed to colorimetric sensors in a reaction chamber. COMSOL Multiphysics analysis was employed to simulate the velocity field of VOCs in the reaction chamber design. The curvature and the position of the baffle in the chamber had a substantial effect on the distribution of VOCs. Through repeated simulation, this study determined that when the baffle curvature is 3.1 and the vertical distance between the baffle front end and air inlet is 1.6 cm, the olfaction visualization system may obtain optimal results. It means that the RGB values change observed in VOCs exposure to sensors would obtain best performance in the optimized system. 

Furthermore, this optimized olfaction visualization system successfully discriminated and clustered the vinegar samples with different storage times. The identification accuracy of vinegar storage can reach more than 90% through the detection of vinegar VOCs in different storages, coupled with PCA and LDA. Storage time of vinegar was detected and discriminated by the olfactory visual system and vinegar as a representative of liquid food. In this study, one brand of vinegar, aromatic vinegar produced in Zhenjiang, was applied to this new reaction chamber. In further work, we may apply this new reaction chamber to the other brands of vinegar.

## Figures and Tables

**Table 1:** Velocity data of the reaction chambers under different velocity fields.

Sensor	Velocity (m/s) ± SD					
	Large-Curvature	Small-Curvature	Close to Inlet	Far from Inlet	Optimization	Without Baffles
1	0.177 ± 0.03	0.029 ± 0.00	0.133 ± 0.02	0.102 ± 0.01	0.108 ± 0.01	0.214 ± 0.05
2	0.170 ± 0.03	0.031 ± 0.00	0.127 ± 0.02	0.098 ± 0.01	0.107 ± 0.01	0.201 ± 0.04
3	0.201 ± 0.04	0.029 ± 0.00	0.151 ± 0.02	0.121 ± 0.01	0.125 ± 0.02	0.239 ± 0.06
4	0.192 ± 0.04	0.030 ± 0.00	0.144 ± 0.02	0.116 ± 0.01	0.122 ± 0.01	0.219 ± 0.05
Mean ± SD	0.185 ± 0.035	0.030 ± 0.001	0.139 ± 0.019	0.109 ± 0.012	0.116 ± 0.013	0.218 ± 0.049

**Table 2:** Classification results by LDA in different reaction chambers %.

PCs	New Reaction Chamber		The Free Gas Volatile Reaction Chamber	
	The Training Set	The Prediction Set	The Training Set	The Prediction Set
1	82.5	85	55	45
2	92.5	90	82.5	70
3	92.5	90	80	75
4	92.5	90	85	65
5	97.5	90	90	65
6	95	90	87.5	65
7	95	90	87.5	70
8	95	95	87.5	70
9	95	90	95	85
10	95	90	97.5	90

## Data Availability

The study did not report any data.

## Supplementary Materials

The following are available at https://www.mdpi.com/2304-8158/10/3/532/s1. Table S1: Response values of colorimetric dyes exposure to Zhenjiang aromatic vinegar with different ages.
