# Supplementary material for: Simulation and Non-Invasive Testing of Vinegar Storage Time by Olfaction Visualization System and Volatile Organic Compounds Analysis
Source: Foods. 2021 Mar 4;10(3):532. doi: 10.3390/foods10030532 (PMC8000387; doi:10.3390/foods10030532)
Supplement: Supplementary file 1 [file foods-10-00532-s001.pdf]

foods

Article-Supplementary Materials

Simulation and Non-Invasive Testing of Vinegar Storage Time by Olfaction Visualization System and Volatile Organic Compounds Analysis

Hao Lin, Jinjin Lin, Benteng Song and Quansheng Chen *

School of Food and Biological Engineering, Jiangsu University, Zhenjiang 212013, China; linhao@ujs.edu.cn (H.L.); 2221818018@stmail.ujs.edu.cn (J.L.); 2221418032@stmail.ujs.edu.cn (B.S.)
* Correspondence: qschen@ujs.edu.cn; Tel.: +86-136-4610-7948

Table S1: Response values of colorimetric dyes exposure to Zhenjiang aromatic vinegar with different ages

Components	CSA	Vinegarwithdifferentages		
		1year	2years	3years
△R	S1	2.04±0.82	0.89±0.92	1.65±0.53
	S2	1.41±1.06	4.39±1.00	5.25±1.13
	S3	1.08±0.79	4.57±0.79	5.60±1.38
	S4	48.67±0.94	48.23±0.89	50.07±1.47
△G	S1	7.48±0.94	9.09±1.29	9.22±1.00
	S2	1.18±0.57	1.80±0.70	2.06±0.66
	S3	1.86±0.45	0.71±0.34	0.69±0.37
	S4	31.98±1.07	30.18±0.57	32.88±1.25
△B	S1	6.83±0.31	6.18±0.94	6.78±1.11
	S2	9.07±1.12	12.78±1.24	11.12±0.61
	S3	9.54±0.87	12.48±1.30	10.77±1.04
	S4	14.26±3.48	18.14±3.13	16.32±1.13
